# Supplementary material for: Evaluation of a bioaerosol sampler for indoor environmental surveillance of Severe Acute Respiratory Syndrome Coronavirus 2
Source: PLoS One. 2021 Nov 15;16(11):e0257689. doi: 10.1371/journal.pone.0257689 (PMC8592464; doi:10.1371/journal.pone.0257689)
Supplement: S1 File — (PDF) [file pone.0257689.s003.pdf]

<30% RH		40-60% RH		>70% RH	
Temperature Mean	23.8	Temperature Mean	25.5	Temperature Mean	24.1
Temperature Median	23.9	Temperature Median	24.9	Temperature Median	24.2
Temperature SD	1.32	Temperature SD	2.07	Temperature SD	0.0427
Temperature IQR	3	Temperature IQR	3.8	Temperature IQR	0.58
Temperature Range	3.88	Temperature Range	6.98	Temperature Range	1.57
Temperature Minimum	21.9	Temperature Minimum	23.1	Temperature Minimum	23.2
Temperature Maximum	25.8	Temperature Maximum	3	Temperature Maximum	24.8
RH Mean	23.5	RH Mean	53.6	RH Mean	35.7
RH Median	23.1	RH Median	54.7	RH Median	35.8
RH SD	3.59	RH SD	3.83	RH SD	1.65
RH IQR	6.8	RH IQR	6.7	RH IQR	2.3
RH Minimum	18.4	RH Minimum	46.8	RH Minimum	91.9
RH Maximum	29.2	RH Maximum	60.1	RH Maximum	98.3
