# Supplementary figures and images for: Evaluation of a bioaerosol sampler for indoor environmental surveillance of Severe Acute Respiratory Syndrome Coronavirus 2
Source: PLoS One. 2021 Nov 15;16(11):e0257689. doi: 10.1371/journal.pone.0257689 (PMC8592464; doi:10.1371/journal.pone.0257689)

## Short Runs

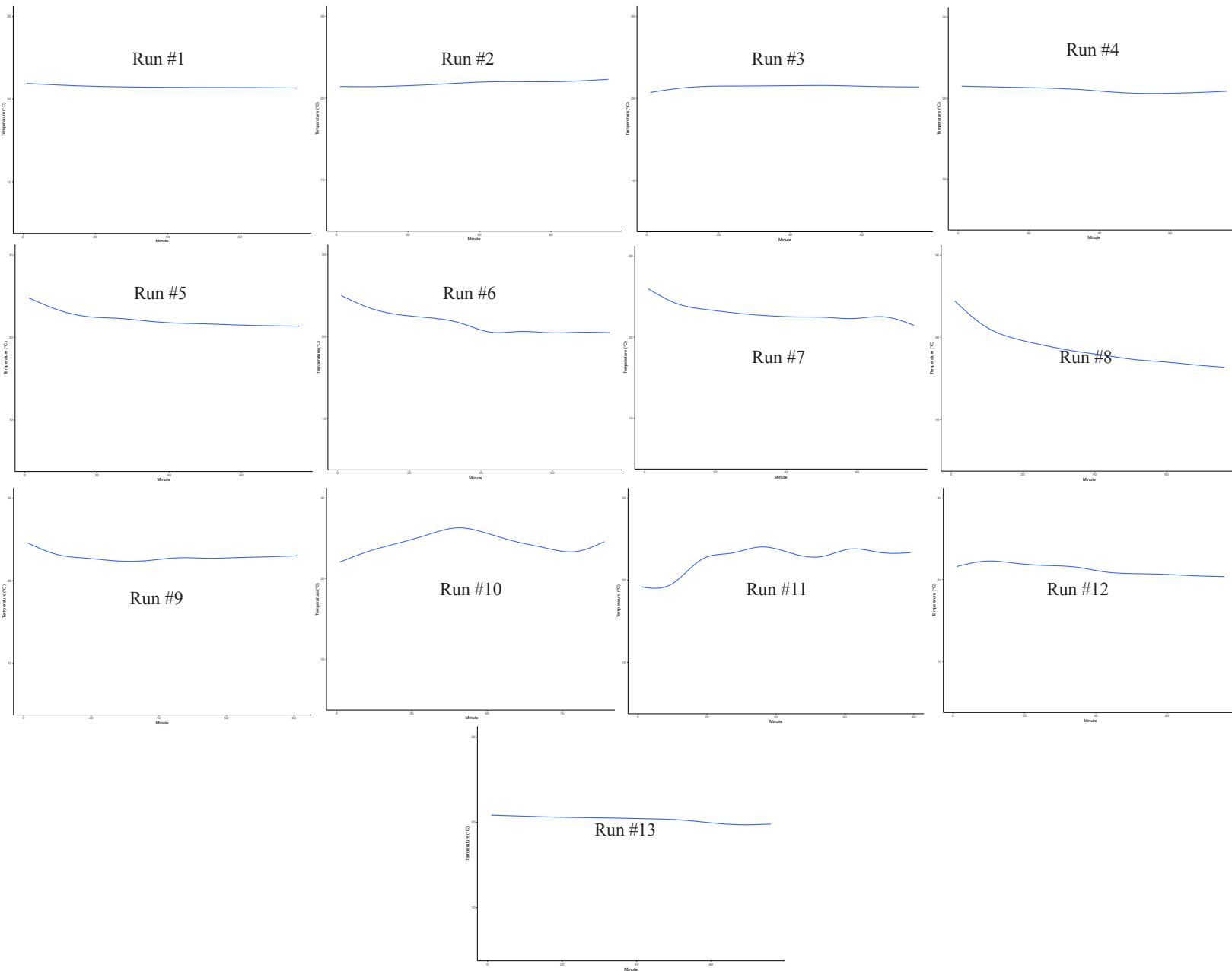

## Long Runs

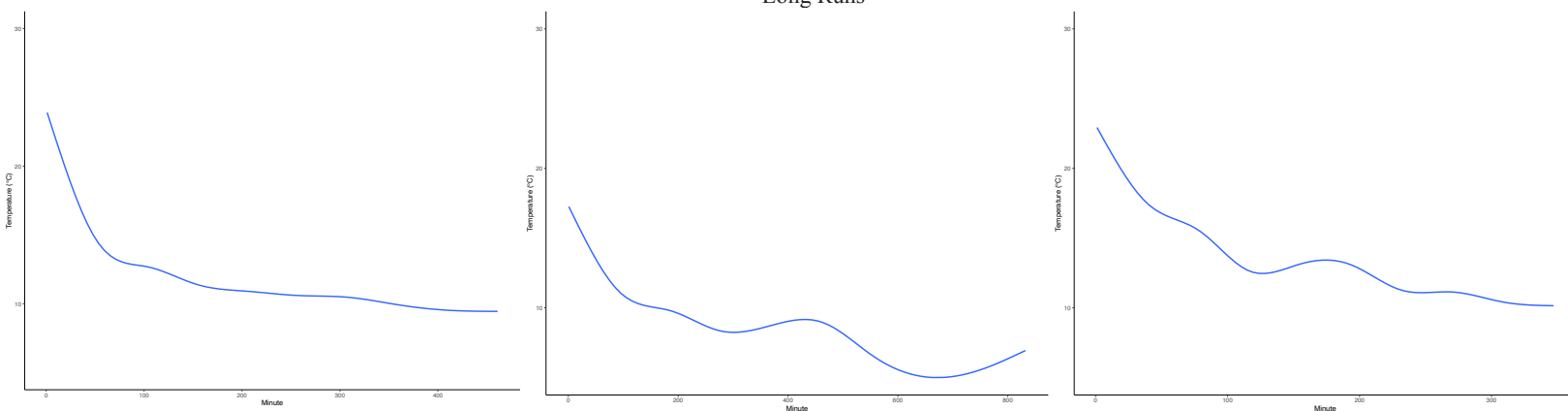

Supplement: S2 File — (PDF) [file pone.0257689.s004.pdf]
